# Metal-organic frameworks as effective sensors and scavengers for toxic environmental pollutants

**DOI:** 10.1093/nsr/nwac091

**Published:** 2022-05-20

**Authors:** Avishek Karmakar, Ever Velasco, Jing Li

**Affiliations:** Department of Chemistry and Chemical Biology, Rutgers University, Piscataway, NJ 08854, USA; Department of Chemistry and Chemical Biology, Rutgers University, Piscataway, NJ 08854, USA; Department of Chemistry and Chemical Biology, Rutgers University, Piscataway, NJ 08854, USA

**Keywords:** metal-organic framework, detoxification, luminescent sensing, pollution, environmental remediation

## Abstract

Metal-organic frameworks (MOFs) constructed from a rich library of organic struts and metal ions/clusters represent promising candidates for a wide range of applications. The unique structure, porous nature, easy tunability and processability of these materials make them an outstanding class of materials for tackling serious global problems relating to energy and environment. Among them, environmental pollution is one aspect that has increased at an alarming rate in the past decade or so. With rapid urbanization and industrialization, toxic environmental pollutants are constantly released and accumulated leading to serious contamination in water bodies and thereby having adverse effects on human health. Recent studies have shown that many toxic pollutants, as listed by the World Health Organization and the US Environmental Protection Agency, can be selectively detected, captured, sequestered and removed by MOFs from air and aquatic systems. Most of these sensing/capture processes in MOFs are quantifiable and effective for even a trace amount of the targeted chemical species. The functional sites (ligands and metals) play a critical role in such recognition processes and offer an extensive scope of structural tunability for guest (pollutants, toxic entities) recognition. Whereas on the one hand, the underlying mechanisms governing such sensing and capture are important, it is also crucial to identify MOFs that are best suited for commercial applications for the future. In this review article, we provide an overview of the most recent progress in the sensing, capture and removal of various common toxic pollutants, including neutral and ionic, inorganic and organic species, with brief discussions on the mechanism and efficacy of selected MOFs.

## Teaser

This review provides an overview on metal-organic frameworks (MOFs) as a promising material class for sensing and capture of toxic pollutants for environmental remediation.

## INTRODUCTION

What is meant by pollution? The introduction of any harmful species into the environment by natural or artificial sources is called pollution. It is fair to say that humans are responsible for most of the pollution taking place in the world at present, due to the rapid growth of population, urbanization and industrialization. These human activities are unavoidable under current circumstances, with many countries pursuing glorification and technological advancements [1]. As a result, a massive surge in environmental pollution has occurred, as seen from environmental reports over the past several decades. Efforts to minimize global environmental pollution are currently underway, to solve the issue or to reduce the effect of pollution, for a greener and sustainable future. Scientists across various domains have contributed significantly to tackling the environmental pollution problem. Waste water and soil are targeted areas because they contain a wide variety of toxic chemicals that are mostly released by industries.

Over the years, several different types of materials, viz. carbon-based materials (graphene, carbon nanotubes, carbon dots, etc.), organic polymers and other inorganic compounds, have shown promising potential in capturing pollutant species from both soil and water [2]. Porous sorbents such as activated carbons, clays, zeolites, porous organic polymers and other ordered mesoporous compounds have also shown their utility in environmental pollution [3,4]. However, several disadvantages, including material stability, high density, lack of structural tunability and low uptake capacity or selectivity, exist in these materials. Metal-organic frameworks (MOFs) are superior adsorbents for the capture of environmental pollutants because of their highly ordered nanoporous structure, high chemical stability and easy processability [5]. The combination of organic struts and metal ions has resulted in over 90 000 well-defined MOF structures being reported so far. These functional building blocks contribute to the precise design of MOFs intended for specific applications that are relevant in the energy and environmental field. The high-density active sites in MOFs and ordered pores allow selective recognition of a target molecule, which is either captured by adsorption in the gas/liquid phase and/or rendered inactive by covalent bonding with active sites and/or photocatalytic degradation [6,7]. Moreover, effective sensing of toxic species is often realized by changes in the fluorescence signals of luminescent MOFs (LMOFs), which results in easy, fast and ppb-level detection of trace-amount contaminants from waste sources [8,9]. MOFs thus could be the next generation of capturing or sensory materials for toxic environmental pollutants. In this review, we give a general overview of the potential of MOFs to capture various pollutants that are found in waste water, radioactive plants and pharmaceutical products that have a direct effect on human health and the environment. In the following sections, we discuss the various mechanisms behind the capture of environmental pollutants (second section), the sensing of ionic pollutants, with a special focus on luminescence-based detection (third section), the detoxification of chemical warfare agents (CWAs) by MOFs (fourth section), and the sequestration of radioactive nuclides, viz. neutral, cationic and anionic species (fifth section). Finally, the capture of toxic organic pollutants is discussed in the sixth section.

## MECHANISMS OF SENSING AND CAPTURE OF ENVIRONMENTAL POLLUTANTS BY MOFS

The rich chemistry of MOFs makes them capable of host–guest interactions dominated by electrostatic, H-bonding, Van der Waals and π-π interactions [10]. As a result, the target pollutant is captured quantitatively by porous MOFs giving a signal output that is measurable by various techniques, including adsorption, optical spectroscopy and X-ray diffraction. Broadly speaking, pollutants found in the environment are classified into (i) neutral, (ii) cationic and (iii) anionic species. Neutral pollutants include toxic gaseous entities such as CO_x_, NH_3_, SO_x_, NO_x_, H_2_S, neutral dyes, volatile organic compounds (VOCs), neutral radioactive wastes, CWAs, polyaromatic compounds, pesticides, herbicides and other persistent organic pollutants. Cationic pollutants include various notorious cations, heavy metal ions, radioactive cations and cationic dyes. Examples of anionic species are toxic oxo-anions, radioactive anions, anionic dyes and other anionic pollutants. In the subsequent sections we give a summary of some of the common neutral and ionic pollutants found in the environment, and the performance and mechanism of sensing and capture by selected benchmark MOFs. We have intentionally excluded the VOCs and purely gaseous pollutants from our discussions because of the wide availability of review articles already present in the literature.

**Figure 1.**
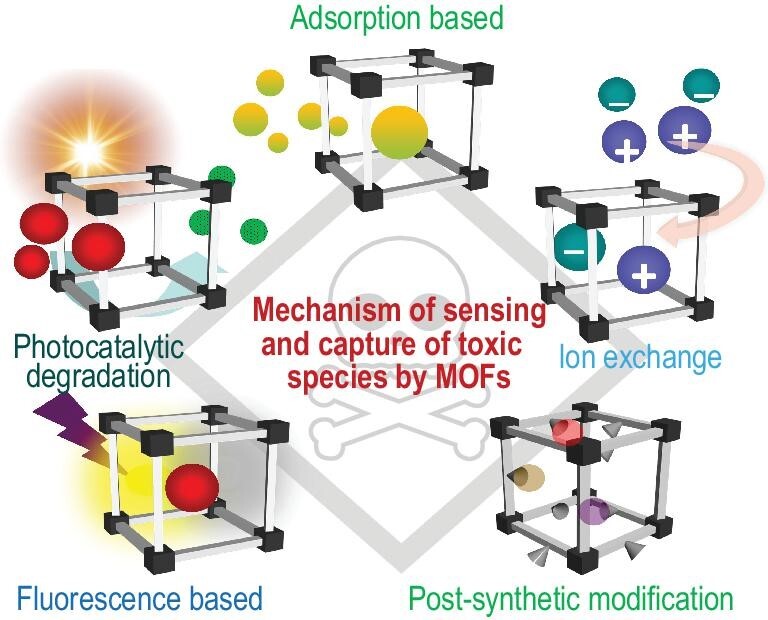
Various mechanisms prevalent in MOFs for the sensing and capture of toxic species.

Mechanistically speaking, the sensing and capture of toxic species by MOFs is based on the mechanisms outlined in Fig. 1.

### Adsorption-based capture

MOFs are porous, with a tunable surface area based on the metal-ligand composition. Thus, shape-specific adsorption of various pollutants occurs in MOFs. In addition, MOFs often exhibit enzyme-like specificity due to their flexible nature, selectively recognizing a particular guest species, and inducing structural changes after adsorption [11]. The high surface area of the MOFs and their size-selective pores have been found to be effective in the capture of various environmental pollutants like CO_x_, NH_3_, SO_x_ and NO_x_. While size effect plays a crucial role in adsorption-based capture, selective diffusion of the analyte as a result of interactions with intrinsically functional active sites in MOFs also plays an important role.

### Fluorometric detection

As discussed previously, LMOFs are an important addition to the subclass of MOFs when it comes to pollutant sensing. Photoemissions involve various mechanisms, including intra-ligand charge transfer (ILCT), metal-to-ligand charge transfer (MLCT), ligand-to-metal charge transfer (LMCT), metal-based emission and guest-induced emission [8,12]. The method used to sense foreign species, such as pollutants, produces changes in the fluorescence output signal by quenching/enhancing the intensity, or by emission energy shifting, giving a direct quantification of the target analyte captured. Also, fast and easy detection of ppb levels makes fluorometric methods of sensing viable for commercialization.

### Capture via ion exchange

Ionic MOFs (iMOFs) are again a subclass of MOFs where there is a residual charge either cationic or anionic on the framework [13]. Since most toxic pollutants found in aquatic systems are ionic, iMOFs have been used extensively for molecular capture via ion-exchange processes. The performance of iMOFs is far superior to some of the common ion-exchange resins, zeolites, layered double-hydroxides (LDHs), etc. because of the added factor of porosity involved in such MOFs. Also, superior kinetics and quantitative exchange of such ionic nuclides makes the ion-exchange process one of the most versatile tools in the sequestration of toxic species.

### Photocatalytic degradation

The application of MOFs to heterogeneous photocatalytic degradation technology involves moderate reaction conditions; low energy demands and a wide application range [14]. The catalytic centres in MOFs are a combination of both photocatalytically active metal ions and electron-rich (π-conjugated) ligands that contribute towards the degradation of various CWAs, dyes and some pharmaceutical products, under visible light. The high catalytic activity at low irradiation time makes MOFs promising semiconducting photocatalysts for pollutant capture and degradation.

### Inactivation by post-synthetic modification (PSM)

PSMs in MOFs are commonly made to incorporate specific functional or recognition sites that can then be used to selectively interact with a particular analyte. Chemically stable MOFs like UiO, ZIF, MIL and PCN series MOFs are often modified through PSM processes to introduce functional groups that can bind either covalently or electrostatically to a toxic species, thus rendering it inactive [15]. This method is very effective for small molecules that can overcome steric factors and readily diffuse into the MOF pores and thereafter bind to the recognition sites.

## DETECTION AND CAPTURE OF IONIC POLLUTANTS BY MOFS

**Figure 2.**
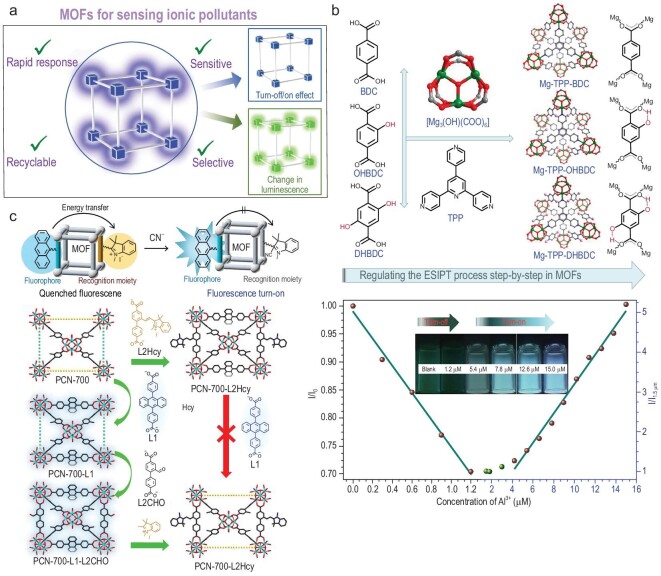
(a) A general schematic of changes that occur in LMOF sensors upon exposure to ionic species. (b) The ESIPT mechanism and interruption by the coordination of Al^3+^ ions. Adapted from ref. [19] with permission from the American Chemical Society. (c) The fluorescent detection of CN^−^ ions through an energy transfer mechanism using a linker installation followed by a post-synthetic modification strategy. Adapted from ref. [22] with permission from Wiley-VCH.

With worldwide urbanization continuously expanding every year, the number of pollutants introduced into the environment increases. These persistent pollutants enter water sources used by billions of people worldwide and can lead to numerous diseases. Thus, the detection and sequestration of toxic pollutants is crucial for the general health of the public. Current technologies for the detection of ionic pollutants include a variety of expensive analytical techniques that make real-time and rapid detection of pollutants challenging. Photoluminescent spectroscopy has several advantages (e.g. it has a lower cost, and is effective and portable) and has attracted great attention in recent years (Fig. 2a) [16]. MOFs have seen significant growth as a platform for sensing and capturing environmental pollutants by virtue of their tunable pore chemistry, high surface area and fast adsorption kinetics. Currently, the most common method for detecting ionic pollutants with MOFs is photoluminescence, where interactions between the pollutants and a MOF alter luminescent properties. Furthermore, capture relies on tailoring pore chemistry to optimize host–guest interactions and maximize loading capacity.

### Luminescent detection of ionic pollutants

Studies have shown that LMOFs are effective sensors for numerous ionic pollutants, including metal ions and inorganic anions. Yang *et al*. reported a dual emission, ratiometric fluorescent Eu-MOF and quantum dot (QD) composite for sensing Hg^2+^ ions [17]. The sensor can detect Hg^2+^ ions with a detection limit of 0.12 nM. Perumal *et al*. designed an ultrasensitive turn-off MOF/fluorophore composite [18]. The design strategy exploits the selective cleavage of a fluorescent Y-shaped DNAzyme comprised of three different DNAzymes. The cleaved DNAzymes then interact with an MoS_2_ functionalized MOF (MOF-MoS_2_NB) that undergoes a fluorescence resonance energy transfer (FRET) based quenching mechanism. For Hg^+^, the detection limit is 0.11 nM. Detection occurs through quenching after direct interaction with the azo group, disrupting electron delocalization. Zhai *et al*. reported a Mg-MOF being used for the selective detection of Al^3+^ ions through interruption of an excited-state intramolecular proton transfer (ESIPT, Fig. 2b) [19]. The MOF is constructed from 2,5-dihydroxybenzene-1,4-dicarboxylic benzoic acid (DHBDC) and a neutral nitrogen linker (TPP). The material shows a unique turn-off then turn-on response to Al^3+^ ions depending on the concentration of Al^3+^ ions. The turn-off response occurs in the range of 0–1.2 μM, while the turn-on response is in the range of 1.2–4.2 μM. Strong binding of Al^3+^ ions to the hydroxyl functional groups of the MOF prevents ESIPT from occurring (Fig. 2b). The MOF has a limit of detection of 28 nM, one of the best reported. Chen *et al*. reported an isoreticular series of MOFs based on linker modification through Schiff-base reactions [20]. The backbone, 2-aminoterepthalic acid, was reacted with salicyaldehyde (SA) derivatives. The resulting imide bond and nearby hydroxyl group serve as a selective interaction site for copper ions that blocks ESIPT. The limit of detection for IRMOF-3a was calculated to be 135 pM. Electron or energy transfer were deemed to be the detection mechanism. Wang *et al*. reported a luminescent cationic lanthanide-based MOF for the detection of Cr_2_O_7_^2−^ ions [21]. Selective detection of Cr_2_O_7_^2-^ occurred instantly with a low limit of detection (LOD) value of 0.56, 1.75 and 2.88 ppb in deionized water, seawater and lake water, respectively. A quenching constant of 3.3 × 10^4^ M^–1^ was reported. The quenching efficiency of this material was attributed to a strong energy transfer, excitation light absorption and rapid uptake. Zhou *et al*. reported post-synthetically-modified PCN-700 for the detection of CN^–^ anions [22]. PCN-700 was grafted with hemocyanine, which serves as a sensitive binding site for cyanide ions. The coordination of CN^–^ to hemicyanine inhibits energy transfer and results in a luminescent turn-on effect with a limit of detection of 0.05 μM, among the best for detection of CN^–^ ions (Fig. 2c).

### Capture of ionic pollutants

The capture of heavy metal ions from solution can be achieved by incorporating functional groups that can react with heavy metal ions via hard-soft acid base (HSAB) interactions. Pardo *et al*. demonstrated the selective capture of mercury using a thioether-decorated bioMOF [23]. Single-crystal structures of the MOF were solved after loading mercury ions to elucidate direct interactions between the thioether chains and mercury ions (HgCl_2_ and CH_3_HgCl), the two most common forms of mercury. The work represents the first account of CH_3_Hg^+^ capture in a MOF and the highest HgCl_2_ loading at the reported date, reducing Hg^2+^ levels from the 10 ppm to 5 ppb range. More recently, Gu *et al*. reported Zr-MSA with a high density of alkyl thiol groups that scavenge for Hg^2+^ ions [24]. The MOF is robust, recyclable and has an uptake capacity of 734 mg g^–1^, with fast adsorption kinetics (<5 minutes). It can reduce the concentration of Hg^2+^ from 10 000 ppb to 0.11 ppb (99.99% capture). Zhong *et al*. reported the post-synthetic modification of MOF-808 with ethylenediaminetetraacetic acid (EDTA) to capture a wide range of metal ions [25]. The soft and hard basic sites result in forced interactions between the framework and all metal ions, resulting in a recyclable heavy metal ion trap capable of removal efficiencies of >99% for 22 different metal ions. The breakthrough experiment confirms the simultaneous removal of 19 heavy metals to the ppb level (below WHO standards). Cai *et al*. reported an Fe-gallic acid MOF for the capture of Cr(_VI_) ions (Cr_2_O_7_^2–^) [26]. Langmuir maximum adsorption capacities show that this material has a potential uptake capacity of 1709.2 mg g^–1^. The mechanism of capture was shown to be chemisorption, ion exchange process and reduction of Cr(VI) into Cr(III). Table 1 lists selected MOFs for the sensing of ionic pollutants [27–53].

**Table 1.** List of selected MOFs for the sensing of ionic pollutants.

MOF	Analyte	Limit of detection (LOD)	Reference
Eu^3+^@UIO-67	Ag^+^	0.049 μM	[27]
MIL-82	Ag^+^	0.09 μM	[28]
Mg-TPP-DHBDC	Al^3+^	28 nM	[19]
Zn-DHNDC	Al^3+^	95 nM	[29]
Eu-MOF	As_3_O_4_^3–^	17.8 nM	[30]
ZIF-90	CN^–^	2 μM	[15]
PCN-700-L_1_-L_2_Hcy-75%	CN^–^	0.05 μM	[22]
[(Cd_3_L_2_)]·(solvent)_x_	Cr_2_O_7_^2–^	4.83 ppb	[31]
Acf@bio-MOF-1	Cr_2_O_7_^2–^	0.25 μM	[32]
[Eu_2_L_3_(DMF)_3_]·2DMF·5H_2_O	Cr^3+^	75.2 nM	[33]
JXUST-3	Cr^3+^	0.049 μM	[34]
[(Cd_3_L_2_)]·(solvent)_x_	CrO_4_^2–^	2.84 ppb	[31]
[[(CH_3_)_2_NH_2_]_3_(In_3_L_4_)]·(solvent)_x_	CrO_4_^2–^	0.52 ppb	[35]
[[Cu^I^_2_(ttpa)_2_][Cu^II^ (bptc)]3·H_2_O·DMF]_n_ (1·3·H_2_O·DMF)	Cu^2+^	0.062 μM	[36]
BPEI-CQDs/ZIF-8	Cu^2+^	80 pM	[37]

## CAPTURE AND DETOXIFICATION OF CHEMICAL WARFARE AGENTS BY MOFS

**Figure 3.**
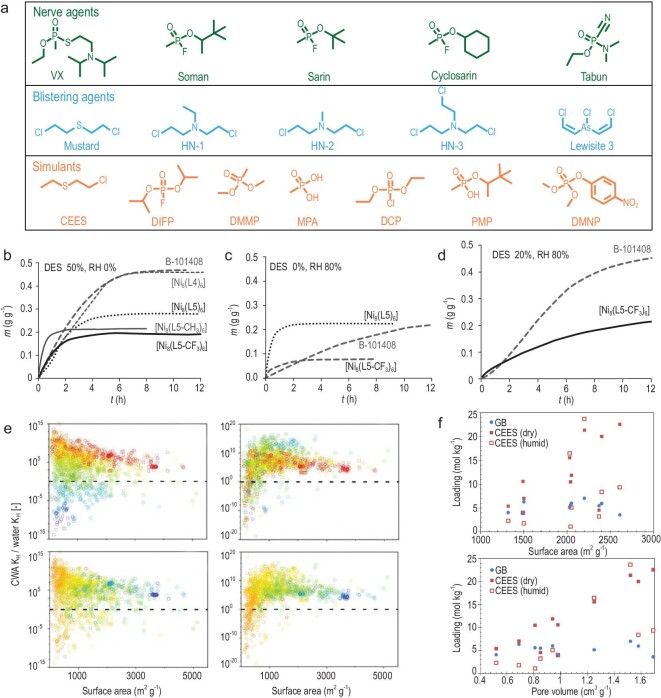
(a) A list of CWA agents including nerve agents, blistering agents and simulants used in laboratory settings. (b) The adsorption profile for DES under no humidity, and (c) no DES and 80% humidity. (d) Mixture of 20% DES and 80% relative humidity (RH) humidity for nickel pyrazolate MOFs. Adapted from ref. [57] with permission from Wiley-VCH. (e) A plot of the surface area vs. selectivity of CWA compared to water for a library of 1647 MOFs. Adapted from ref. [60] with permission from the American Chemical Society. (f) A plot of the uptake capacity for CWA compared to the surface area (top) and pore volume (bottom) under dry and humid conditions. Adapted from ref. [62] with permission from the American Chemical Society.

Chemical warfare agents (Fig. 3a) are an extremely dangerous class of chemicals that are purposefully used to harm exposed individuals. Although they are banned by the Chemical Weapons Convention (CWC), there have been an alarming number of reports in recent years. Thus, it is crucial to development methods to protect individuals from chemical-warfare-agent (CWA) exposure, either through direct capture, catalytic degradation or detection. This section of the review will focus on work directed towards the adsorption of CWAs, rather than catalytic decomposition as summarized elsewhere [54]. MOFs are attractive candidates for the adsorption of CWAs because of their tailorable chemical tunability, which promotes strong guest–host interactions. The two main types of modern CWAs are nerve agents and blistering agents [55]. Nerve agents work by inhibiting the enzyme acetylcholinesterase, which results in a lack of control over muscular contractions leading to cardiac arrest or asphyxiation within seconds.

One of the earliest studies on the adsorption of CWAs was reported by Navarro *et al*. using a Zn-imidazole MOF [Zn_4_(μ_4_-O)-(μ_4_-4-carboxy-3,5-dimethyl-4-carboxy-pyrazolato)_3_] [56]. The calculated heat of adsorption—50.1 kJ mol^–1^, 44.8 kJ mol^–1^ and 17.9 kJ mol^–1^ for dethylsulfide (DES), diisopropylfluorophosphate (DIFP) and water, respectively—showed preferential binding towards CWAs under competitive conditions. The uptake capacity for DIFP was reported to be 60 mg g^–1^. Shortly after, in 2013, Navarro *et al*. reported seven isoreticular [Ni_8_(L_n_)_6_] pyrazolate-based MOFs with varying linker modifications for the selective adsorption of CWA simulants (Fig. 3b–d) [57]. The -CF_3_ functionalized framework, [Ni_8_(L5-CF_3_)_6_], demonstrated the highest hydrophobicity (low water uptake ∼0.1 m/g g^–1^) and best competitive adsorption versus water (20% DES, 80% RH). Meanwhile, the isoreticular analogue [Ni_8_(L4)_6_] had the highest uptake capacity in dry conditions (∼0.48 m/g g^–1^). Heat of adsorption values were calculated to be 65.87 kJ mol^–1^ for [Ni_8_(L5-CF_3_)_6_]. Peterson *et al*. reported linker modifications to UiO-66 to improve adsorption of CWA chlorine gas [58]. UiO-66-NH_2_ shows significant retention of Cl_2_ in micro breakthrough plots compared to UiO-66. The proposed mechanism for Cl_2_ capture is electrophilic aromatic substitution. A removal capacity of 1.24 g of chlorine gas per gram of UiO-66-NH_2_ was reported. Johnson *et al*. reported a family of functionalized UiO-67 derivatives that can be used for the adsorption of dimethyl methylphosphonate (DMMP) [59]. Initial calculations for the interaction between DMMP and functional groups follow the trend -NH_2_ > -CH_3_ > -H. The three MOFs were synthesized and studied via temperature programmed desorption (TPD) to determine the strength of the interactions. TPD measurements coincide with the calculated adsorption affinities. Fairen-Jimenez *et al*. reported a high-throughput molecular simulation screening of ∼3000 MOFs. Filtering by size limitations, hydrophobicity and calculated uptake capacities >4 mol kg^–1^ reduced the number to three MOFs for sarin, soman and mustard gas (Fig. 3e) [60]. [Ni_3_(BTP)_2_] was then synthesized and shown to have an uptake capacity of 0.6 mol kg^–1^, with a DES concentration of eluted gas of 0.05 ppm.

Zirconium-based MOFs are well studied for the hydrolysis of CWAs and are known to have strong interactions between the Zr_6_ secondary building unit (SBU) and CWAs. Mandal *et al*. showed that NU-1000 and UiO-67 have strong adsorption affinities for both CEES and DMMP [61]. The adsorption rates and amounts for both of these simulants was monitored through Inductively Coupled Plasma-Atomic Emission Spectroscopy (ICP-AES) and reveal that NU-1000 outperforms UiO-67 in the uptake capacities of both CEES and DMMP, with values of 4.197 mmol g^–1^ and 1.70 mmol g^–1^ compared to UiO-67 values of 4.000 mmol g^–1^ and 0.90 mmol g^–1^, respectively. Diffuse reflectance infrared fourier transform spectroscopy (DRIFTS) experiments show that CEES and the Zr-MOFs experience hydrogen bonding between the chloro and thioether groups, while DMMP uptake occurs at the Zr node. Farha *et al*. report the role topology plays with respect to the capture and reactivity of CWAs [62]. The study outlines the use of 10 different Zr-based MOFs with varying SBU connectivity, topology, pore functionalization and open metal sites. Breakthrough experiments on sarin and CEES reveal that frameworks with higher surface areas and large pores typically result in higher uptake capacities for sarin and CEES (Fig. 3f). Furthermore, MOFs with -OH- and -H_2_O-decorated SBUs have higher uptake capacities in humid environments. Computational modelling serves as a powerful tool to predict interactions between MOF frameworks and CWAs. While strictly computational work is not discussed here in detail, we direct our readers to several key studies for the design of next-generation CWA sorbents [63,64].

## SEQUESTRATION OF RADIONUCLIDES BY MOFS

With the advancement of nuclear energy and industry, radioactive by-products and wastes have seriously contaminated the marine environment, soil, groundwater, etc. and have caused a great deal of environmental concern. Moreover, the radioactive toxicity associated with such species causes grave human health hazards including diseases like nephrotoxicity, immune system disorders, cancers and cardiovascular diseases. Current methods of storing these millions of gallons of radioactive waste species include storage in geological repositories, mined repositories or sub-surface storage waste facilities. However, leakage from the waste tanks could potentially lead to contamination of ground water, and would affect the safety of public life and the environment. Thus, capture, storage and possible sequestration of such radioactive nuclides is of paramount importance. Radioactive waste is typically classified as either low level (LLW), intermediate level (ILW) or high level (HLW), and depending on the degree of radioactivity, appropriate measures must be taken for disposal and sequestration. Porous sorbents like MOFs could be a potential game changer in this respect due to their exceptional porosity, high adsorption ability and physiochemical stability. Their fast adsorption kinetics, high adsorption capacity and pore-surface functionalization make MOFs the ideal candidates for storage and sequestration of toxic species.

### Capture of neutral radioactive pollutants

**Figure 4.**
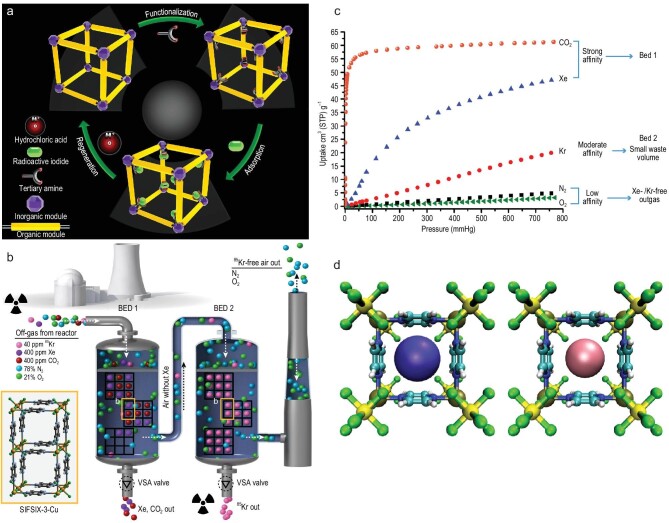
(a) Molecular traps incorporated in MIL-101 for capturing organic iodides from nuclear waste. Adapted from ref. [68] with permission from Springer Nature. (b and c) Two-bed breakthrough set-up for removal of competing gases CO_2_ and Xe, and thereby selective capture of Kr over N_2_ and O_2_, and adsorption isotherm stating the same. (d) Theoretically predicted structures of Xe (violet) and Kr (pink) in a SIFSIX-3-M MOF. Adapted from ref. [73] with permission from Springer Nature.

The radioactive fission of ^235^U in nuclear reactors results in the formation of ^129^I, which has a considerably long half-life (t_1/2 _= 1.57 × 10^7^ years), a certain amount of ^131^I (half-life of 8.02 days) and some other radioactive organic iodides (ROIs) as well [65]. In one of the earlier reports, Sava *et al*. demonstrated the chemisorption of molecular iodine (I_2_) by zeolitic imidazolate framework-8 (ZIF-8) cages. The iodine molecules were located crystallographically inside the porous cages of ZIF-8; there were up to 5.4 molecules of I_2_ per cage of ZIF-8 [66]. Pore functionalization in the MIL-53 series was found to be very effective for capturing iodine from cyclohexane. It was shown that amino-containing MOFs like MIL-101-NH_2_, CAU-1 or MIL-53-NH_2_ had the highest capture of iodine compared to the unsubstituted MOFs, which proved that an optimal balance of porosity and charge transfer between the electron-rich substituents (like -NH_2_) and I_2_ is the key parameter for high iodine uptake [67]. Li and co-workers discovered that pore-surface functionalization, achieved by grafting tertiary amine groups in MIL-101(Cr), resulted in one of the best candidates for capturing organic iodides viz. methyl iodide (CH_3_I) at high temperature [68]. MIL-101-Cr-TED and MIL-101-Cr-HMTA (TED = triethylenediamine and HMTA = hexamethylenetetramine) were employed as molecular traps for ROI capture (Fig. 4). The adsorption capability of these functionalized MOFs was experimentally determined to be 160 and 174 wt% at 30°C, and at a high temperature of 150°C showed 48 and 39 wt% capture of organic iodides. These MOF materials showed exceptional recyclability and repeatability via simple washing with acidic solution. Schroder and his team members exhibited halide–halide interactions in MFM-300 (M = Al^3+^, Sc^3+^, Fe^3+^, In^3+^) for high uptake of I_2_ [69]. The self-aggregation via MOF-I_2_ and I_2_-I_2_ resulted in the formation of a triple helical chain, which in turn was responsible for an I_2_ storage density of 3.08 g cm^−3^ in the MFM-300 (Sc) MOF.

The processing of used nuclear fuel (UNF) generates a mixture of noble gases like ^85^Kr, ^127^Xe and ^129^I. MOFs have emerged as potential materials for the adsorption of such gases due to their structural tuneability, high selectivity and radiation-resistant nature. Since Xe is more polarizable than Kr, MOFs show higher selectivity towards Xe. Banerjee *et al*. used a computational screening approach to predict that calcium-based nanoporous MOF, SBMOF-1, was one of the most effective MOFs for Xe adsorption. Column breakthrough experiments conducted at room temperature showed a high uptake capacity of 11.5 mmol kg^–1^ even in the presence of water vapour [70]. The high uptake capacity of this MOF was attributed to the optimal pore size and the dense wall of the MOF structure. The pore size and geometry of MOFs were further explored in order to tune the adsorption behaviour in SIFSIX-3-M series MOFs [71]. The Ni variant showed a pronounced inflection during Xe adsorption, due to the rotational behaviour of the aromatic rings triggered by the sorbate–sorbent interactions. Such ‘breathing’ behaviour in MOFs, which is intended to selectively capture Xe, was one of the prime examples of how the structural tuneability in MOFs could be useful in capturing such radioactive species from reprocessing facilities. A squarate-based polar hydroxy (-OH) decorated MOF was found to exhibit record-high Xe/Kr separation and Xe uptake capacity at ambient temperature and pressure. In this study, the addition of polar functional groups coupled with exact-fit pore structure was the key factor for such high selective capture of Xe over a mixture of other gases [72]. The quest for ^85^Kr-adsorbing MOFs led to the systematic study of SIFSIX-3-M (M = Zn, Cu, Ni, Co or Fe) by Elsaidi *et al*. [73] using a two-bed breakthrough method. The selectivity order was evaluated to be Xe, CO_2 _> Kr > N_2_ and O_2_. As shown in Fig. 4b and c, the first bed showed high selectivity and removal of Xe and CO_2_ whereas the second bed was responsible for the selective capture of Kr over N_2_ and O_2_. Moreover, SIFSIX-3-M MOFs were stable upon exposure to γ irradiation, among which SIFSIX-3-Cu was found to be most stable even after 50 kGy radiation.

### Capture of cationic radioactive pollutants

**Figure 5.**
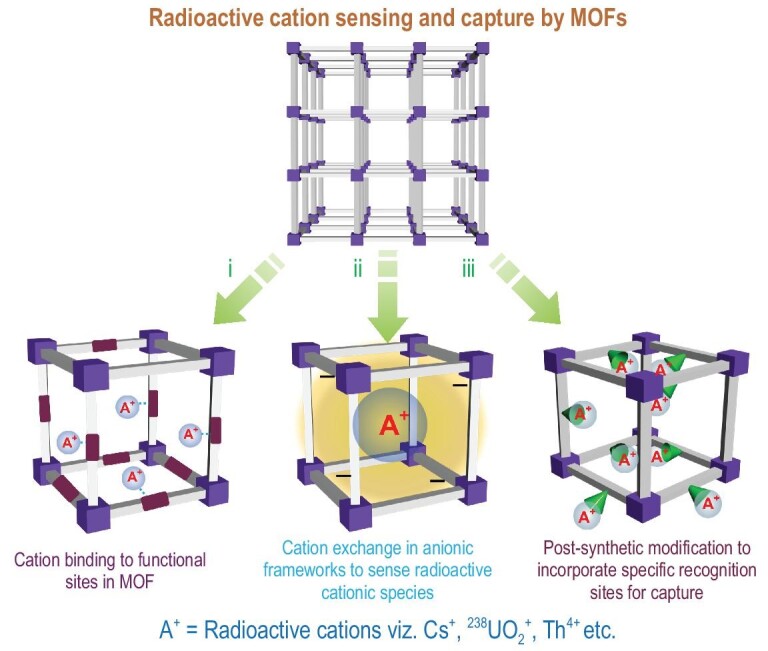
Mechanisms for the capture of various cationic radioactive pollutants by MOFs.

Cationic radioactive pollutants like ^137^Cs^+^, ^90^Sr^2+^, ^232^Th^4+^ and ^238^UO_2_^+^ are the most common source of charged waste found in nuclear processing units. Uranium is one of the most important fuels in nuclear power plants and occurs mostly in nature as ^234^U, ^235^U and ^238^U, of which ^235^U and ^238^U are the two elements most widely used in nuclear fission materials. Although it is such an important fuel, uranium causes severe environmental and human health hazards. Uranium exists in nature in various oxidation states, viz. U^3+^, U^4+^, U^5+^ and U^6+^, out of which the most stable form is U^6+^. It exists in a stable cationic form, UO_2_^2+^ (uranyl), which readily forms salts with various oxygen donor ligands. Compared to uranium, thorium, which mostly exists as Th(IV), is less toxic. However, due to its large abundance and role as surrogate cation for Np(IV) and Pu(IV), knowledge about its chemical properties and possible capture is important for radioactive waste management. Extraction and sequestration of such toxic cationic species is of paramount importance because of their high chemical reactivity and radiological activity. Due to the chemical tuneability in MOFs, they can be deemed as efficient scavengers for toxic cationic species. The mechanism involved in the recognition and capture of such toxic species usually involves: (i) coordination of functional groups in the MOF framework with the toxic entity, (ii) cation exchange in charged frameworks and (iii) a post-synthetic modification strategy to impregnate binding sites in MOFs for selective recognition and capture (Fig. 5). Host–guest interaction involving hydrogen bonding interactions, Lewis acid/base chemistry and size exclusion play a pivotal role in governing the uptake capacity of such toxic species in the MOF framework.

Cation exchange was employed in an anionic uranyl framework containing disordered [NH_2_(CH_3_)_2_]^+^ [74]. Fast and efficient capture of Cs^+^ was achieved with a high sorption amount of 145 mg g^–1^ from aqueous solutions within 20 minutes. Such a superior performance makes this one of the best-known MOFs for Cs^+^ capture. Wang demonstrated the ultrahigh capture of ^90^Sr^2+^ from both seawater and acidic solutions using an anionic MOF, SZ-4 [75]. The capture process was monitored crystallographically by solid-state transformation, which gave insight into the mechanistic details of the two-step intercalation process. The collective effect of coordinating the Sr^2+^ to the oxide layer and the dimethylamine interlayer was proposed to be the governing factor behind such efficient capture.

In one of the very first reports of uranyl capture by MOFs, Lin and co-workers demonstrated that UiO-68-topology-based phosphorylurea-derived MOFs were efficient scavengers of uranyl ions [76]. The sorption amount was 217 mg Ug^–1^ from artificial seawater. The spatial structure in superb-uranyl binding protein (SUP) was mimicked by fabricating a 4-aminoisophthalic-acid-incorporated UiO-66 MOF (UiO-66-3C4N). The nanopockets in UiO-66-3C4N, containing carboxyl and amino groups, exhibited a superior binding capacity of uranyl ions. The uptake capacity of uranyl ions from natural seawater was estimated to be 6.85 mg g^–1^, which was the highest reported capture in the domain of microporous materials [77]. Fast and easy detection of uranyl ions was achieved by a luminescent anionic MOF, [Tb(BPDC)_2_]·(CH_3_)_2_NH_2_ (DUT-101), containing protonated dimethylammonia molecules. The sensing phenomena was attributed to the intermolecular electron

transfer and resonance energy transfer resulting in selective fluorescence quenching in the presence of uranyl ions [78]. The exposed Lewis basic sites in a terbium-based MOF showed a luminescence quenching effect in the presence of uranyl ions. The detection limit was calculated to be 0.9 μg/L, which is far below the permissible limit set by the United States Environmental Protection Agency.

For Th^4+^, it has been observed that the increase of -COOH groups in the UiO-66 structure improved the sorption kinetics, capacities and selectivity. This is attributed to the binding of the Th^4+^ ions to the free -COOH functional groups in the structure [79]. Post-synthetic modification in a MnSO-MOF, resulting in selective demetallation of the Mn^2+^ ions, resulted in the selective capture of Th(IV) from Ln(III) ions [80]. The sorption capacity was estimated to be 46.345 mg of Th/g and was a result of the coordination of the Th^4+^ ions with the chelating atoms of the salen ligand. A Eu^3+^-based MOF with the molecular formula [Eu_2_(MTBC)-(OH)_2_(DMF)3(H_2_O)_4_]·2DMF·7H_2_O, (ThP-1), MTBC^4–^ = [1,10-biphenyl]-4-carboxylate)] showed distinct fluorescence response over a wide range of concentrations. The rationale behind such a selective response towards Th^4+^ was proposed to be LMCT in a Eu^3+^–OH–Th^4+^ complex formed upon incorporation of the Th^4+^ into the MOF [81].

### Capture of anionic radioactive pollutants

**Figure 6.**
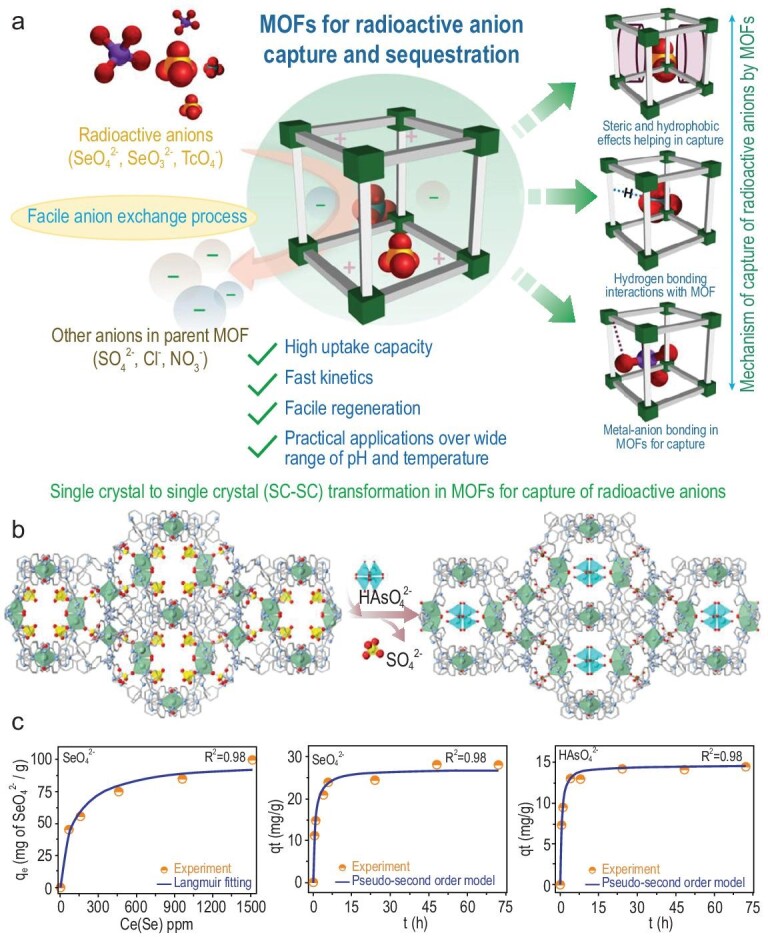
(a) Mechanisms for the capture of various anionic radioactive pollutants by MOFs. (b) Anion exchange as a versatile tool for radioactive sequestration of toxic HAsO_4_^2–^ in iMOF-C. (c) Quantitative removal of Se^VI^ (SeO_4_^2–^) and As^V^ (HAsO_4_^2–^) from water by the same MOF. Figure 6b and c are adapted from ref. [86] with permission from Wiley-VCH.

Some of the most common notorious anionic radioactive elements include ^99^TcO_4_^–^, ^79^SeO_4_^2–^ and ^79^SeO_3_^2-^ [82]. For capture of such hazardous radioactive anionic pollutants the most common strategy employed in MOFs is anion exchange (Fig. 6). Cationic frameworks containing residual negative charge are often exchanged with such perilous anions to incur structural, optical and other physical/chemical changes in the MOF material. The incoming anions result in suitable signal transduction across the host framework by means of suitable host–guest interactions dominated by hydrogen-bonding interactions that result in selective capture of such toxic species. ^99^TcO_4_^–^, which is a highly water soluble and mobile anionic residue found in glass waste at high temperature, needs to be sequestered before glassification. Ion exchange in MOFs is the most versatile method of capturing such toxic ^99^TcO_4_^–^ from waste water, nuclear waste streams, etc. by virtue of a specific interaction with the host framework. Also, inherent hydrophobicity of MOFs can facilitate prompt capture of the anion, as shown in SCU-102 [(Ni_2_(tipm)_3_(NO_3_)_4_)] [83]. The MOF showed record-high ^99^TcO_4_^–^ uptake selectivity in the presence of NO_3_^–^ and SO_4_^2–^. Quantitative removal was achieved with very fast sorption kinetics and a high distribution co-efficient, which are the key parameters for the use of this material for practical applications. The uptake capacity was estimated to be 291 mg·g^–1^, which is the highest reported level of ^99^TcO_4_^–^ capture by inorganic materials.

The supreme performance of this material was attributed to the efficient binding of the anion in the hydrophobic pockets of the MOF, which was calculated by DFT. *In situ* polymerization of ionic liquids inside the pores of MIL-101(Cr) was employed to serve a dual-purpose strategy: (i) to incorporate ionic filler, which acts as active sites, and (ii) to add a protective outer coating to the MOF, which enables superior performance of the composite material even under extreme conditions (6M HNO_3_). The intrinsic hydrophobicity of the MOF pores also affords high selectivity towards pertechnetate ion even in the presence of other competing anions like NO_3_^–^, Cl^–^ and NO_2_^–^, which are present in larger quantities (almost 300 times the amount) in a simulated Hanford LAW melter recycle stream [84]. Removal of ^99^TcO_4_^–^ from a basic nuclear waste tank by MOFs is a challenge due to stability issues and a strong radiation field. Wang and his group members came up with a breakthrough work in which they designed a base stable cationic MOF with high resistance towards β and γ radiations. The MOF, SCU-103, showed a high removal efficiency of ^99^TcO_4_^–^ from alkaline medium. The sterically crowded metal centres in the MOF provided ideal recognition pockets for ^99^TcO_4_^–^ and acted as a shielding agent towards water and alkaline solutions, as confirmed by DFT and molecular simulation results. The electrostatic repulsion caused by the incoming ^99^TcO_4_^–^ causes unbinding of the parent NO_3_^–^ present inside the SCU-103 pores, resulting in a facile anion exchange process [85]. Cation exchange in ionic MOFs has also been employed to selectively capture ^79^SeO_4_^2–^ and As^V^ (HAsO_4_^2–^) in an ionic MOF, viz. iMOF-C composed of 4-(1H-imidazol-1-yl)phenyl)amine ligand and Ni^2+^ cation [86]. The parent MOF had free-lying exchangeable SO_4_^2–^ anions that could be exchanged post-synthetically with such toxic and radioactive anionic species as ^79^SeO_4_^2–^ and HAsO_4_^2-^ (Fig. 6b), resulting in high sorption capacities of 100 mg g^–1^ and 84 mg g^–1^; this was one of the highest reported captures of such perilous anions in the domain of porous materials (Fig. 6c). Crystallographic insights about the anion exchange mechanism also supplemented the fact that favourable interactions like hydrogen bonding, and the geometry of the incoming anions, were essential in such a capture mechanism.

Separation of ^79^SeO_3_^2-^ from a mixture of ^79^SeO_3_^2–^/^79^SeO_4_^2–^ is important as it is far more toxic and radioactive than the latter. Yang and co-workers achieved this property using a Bi-containing MOF, CAU-17, which showed a remarkable selectivity, high adsorption capacity and fast kinetics for ^79^SeO_3_^2−^ anion [87]. A new Bi-O-Se bond formation, which was not seen in the case of ^79^SeO_4_^2-^ anion adsorption, was shown in the adsorption site of the CAU-17 MOF. The high uptake capacity of 255.3 mg g^–1^ and fast kinetics over a wide pH range showed the utility of this MOF for radioactive nuclide sequestration.

## CAPTURE AND SENSING OF ORGANIC ENVIRONMENTAL POLLUTANTS BY MOFS

Persistent organic pollutants (POPs) have caused a serious threat to human health and the environment since their first commercial use in the Second World War. Most of these chemicals are released in the wind or aquatic systems, are persist in the environment and transferred from one species to another through the food chain. In view of this, 90 countries across the globe have signed a United Nations Treaty in Stockholm, Sweden, and have identified 12 POPs (the ‘dirty dozen’) and agreed to limit/restrict their usage due to their mutagenic effects and disruption of the ecological balance [88]. Broadly, POPs are classified into two types: (i) intentionally produced and (ii) unintentionally produced substances.

Pharmaceutical and personal care products (PPCPs), toxic agrochemicals, organic dyes, feed additive sweeteners, etc. all fall under the POP category; rapid industrialization and the growing population have escalated the pollution of aquatic bodies and this needs remediation. Most methods of water purification, like reverse osmosis, membrane separation and advanced oxidation processes (AOPs), are expensive and require huge energy inputs.

MOFs have shown remarkable potential in the capturing and sensing of such perilous organic species, mostly due to their intrinsic porosity, guest responsive functional sites and tuneable architectures. The versatility in their design and stability over a wide range of pH make MOFs usable for practical purposes in the capture of toxic organic pollutants from water. Some well-known organic pollutants and their capture by MOFs are discussed in the subsequent sections.

### Capture and sensing of PPCPs

Huge production of pharmaceutical products like antibiotics, anti-inflammatories and antimicrobials has resulted in the accumulation of huge amounts of waste that causes water pollution to a large extent. Porous adsorbents, more specifically MOFs, could be a solution to the problem of trapping and capturing these PPCPs, due to their ultrahigh adsorption capacity, removal efficiency and low operating costs. Fluorescence sensing and capture of PPCPs by MOFs is an effective way to remove such contaminants due to the very high selectivity and sensitivity governing the processes. For example, two MOFs, BUT-12 and BUT-13 (BUT = Beijing University of Technology), which are composed of Zr^4+^ and  II-conjugated organic ligands, showed very effective capture of 12 antibiotics of 5 different classes, viz. nitroimidazoles (NMs), nitrofurans (NFs), chloramphenicols (CPs), sulfonamides (SAs) and β-lactams. Among them, the nitro (NO_2_) containing antibiotics like nitrofurazone (NZF) and nitrofurantoin (NFT) showed the maximum quenching efficiency (92%–95%), which was a direct result of the energy and/or electron transfer processes occurring in the fluorescence mechanism [89]. With regard to the sensing of antibiotics, MOFs tend to perform well in a wide range of pH, as shown by Zhu *et al*. [90] using the MOF FCS-1, which showed excellent performance in the capture of sulfonamide antibiotics in simulated waste water conditions such as a pH range of 3–9, and also in the presence of other competing heavy metal pollutants. Similarly, a tetraphenylethene (TPE) based MOF, viz. Zn_4_O(BCTPE)_3_, where H_2_BCTPE is 4,4′-(1,2-diphenylethene-1,2-diyl)dibenzoic acid, which showed aggregation-induced emission (AIE) initially, selectively sensed nitrofurazone and metronidazole, resulting in 93% and 50% quenching respectively. The presence of open metal sites in PCN-124-stu(Cu) has also been utilized to selectively capture ofloxacin (OFL), enrofloxacin (ENR) and norfloxacin (NOR), with a remarkable uptake capacity of 198, 292 and 354 mg g^−1^ respectively [91]. Hydroxy- and amino-functionalized MIL-101(Cr) showed good capture of similar pharmaceutical products compared to a nitro-functionalized analogue, due to the hydrogen bonding interactions with the incoming guest molecules. Solid phase extraction (SPE) with MIL-101(Cr) was employed to adsorb sulfadiazine (SDA), sulfamethoxazole (SMX), sulfachloropyridazine (SCP) and sulfamethazine (SMZ) from contaminated water samples, which demonstrated the practical utility of MOFs for the capture of such toxic entities [92].

### Capture and sensing of toxic agrochemicals

**Figure 7.**
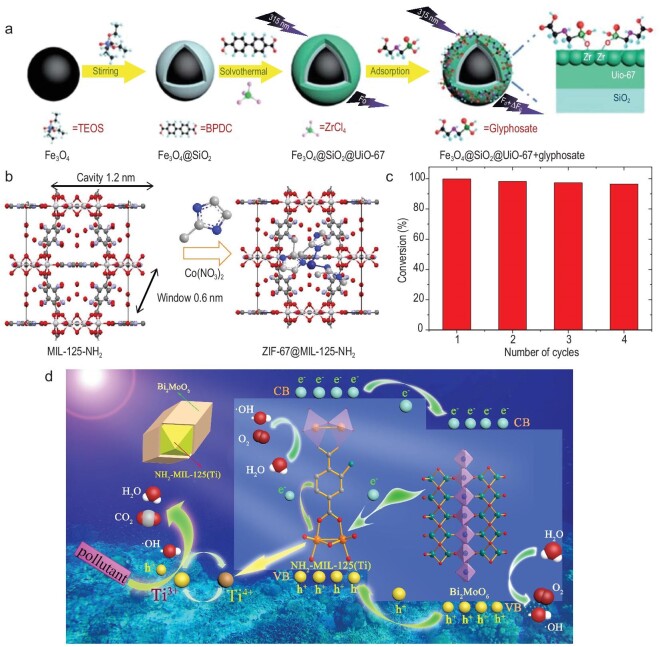
(a) Core-shell heterostructure of Fe_3_O_4_@SiO_2_@UiO-67 showing selective interaction leading to adsorption of glyphosphate. Adapted from ref. [94] with permission from the Royal Society of Chemistry. (b) Preparation of ZIF-67@ZIF-8@MIL-125-NH_2_ and (c) photocatalytic activity of the same composite towards 4-nitrophenol, showing almost 100% removal efficiency. Adapted from ref. [99] with permission from Elsevier. (d) Mechanism of dichlorophenol and trichlorophenol capture by a composite based on NH_2_-MIL-125 and Bi_2_MoO_6_ resulting in 93.28% and 92.19% degradation respectively. Adapted from ref. [100] with permission from Elsevier.

To produce the desired quality and quantity of agricultural products, farmers all over the world use huge quantities of pesticides. The extensive use of herbicides, fungicides, insecticides, molluscicides, rodenticides, bactericides and miticides has detrimental effects on human health. Common pesticides, which include organophosphorus, organochlorines, carbamates, chlorophenols and various other POPs, need to be removed from food products to maintain food safety and human health. MOFs have recently answered some of the outstanding questions in this respect, especially because of their large functional pores, which could be utilized to trap and remove these common pesticides from food. UiO-67 has shown good capture of organophosphates like glyphosate (GP) and glufosinate (GF), 360 and 537 mg g^−1^ respectively [93]. Composites like UiO-67/GO (GO = graphene oxide) and Fe_3_O_4_@SiO_2_@UiO-67 have shown superior recognition and separation of glycophosphate molecules due to a higher density of active sites and the presence of Zr–O clusters that interact specifically with the phosphoric acid groups (Fig. 7a) [94]. In general, the chemical stability of Zr MOFs results in the applicability of such MOFs to the capture of toxic pesticides from aqueous media in various physiological conditions (pH, temperature pressure), and holds great promise in terms of practical application. In general, the adsorption of such pesticides by MOFs usually follows the Langmuir and Freundlich isotherm models, which are denoted by Equations (1) and (2).

(1) \begin{equation*} \frac{{Ce}}{{Qe}} = \frac{1}{{QmKl}}\; + \frac{{Ce}}{{Qm}}, \end{equation*}

(2) \begin{equation*} ln\;Qe\,Qe = \ln Kf + \frac{{lnCe}}{n}\;. \end{equation*}

In the equations, *Kl* (L mg^−1^) denotes the Langmuir equilibrium constant, *Qm* is the maximum adsorption capacity (mg g^−1^) and Kf ((mg g^−1^) (L mg^−1^)1/*n*) and *n* are Freundlich constants.

The fluorescence-based approach using LMOFs has also been utilized in trace-level selective detection and recognition of pesticides. FRET, and/or photoinduced electron transfer (PET) mechanisms usually prevail in such systems. The fast response and low LOD achieved by the fluorescence process make LMOFs ideal candidates for the recognition of pesticides from both aqueous and non-aqueous media. Several organophosphate and organochlorine pesticides (OPPs and OCPs) have thus far been sensed using LMOFs. Parathion, Methyl Parathion, Azinphos-methyl and Chlorpyrifos, which are commonly used OPPs, are highly toxic and need to be removed from food products completely prior to packaging. Several Zn-, Cd- and Zr-based MOFs have shown utility in the detection of such OPPs due to their intrinsic fluorescent nature, and also show high quenching in fluorescence in the presence of such analytes in an aqueous medium. Also, the low LOD levels (ppm or ppb) are in line with the permissible limit set by the Food and Drug Enforcement Agency (FDA). Similarly, another notorious agent, viz. 2,6-dichloro-4-nitroaniline (DCN), which is an OCP, has been detected in very low limits by using luminescent MOFs. Significantly, in all such cases the quenching mechanism involved either FRET or PET or a combination of both. In Table 2, we list some of the common pesticides and their detection performance by either adsorption or fluorescence-based methods using MOFs.

**Table 2.** Performance of well-known MOFs in the capture and sensing of common pesticides.

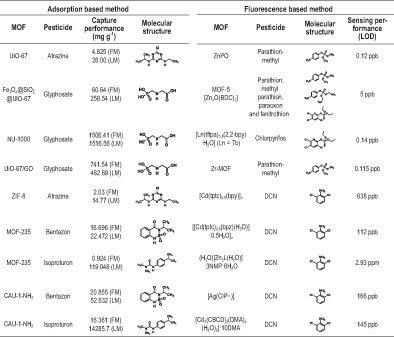

^a^FM = Freundlich model, LM = Langmuir model.

According to the World Health Organization (WHO), the safe limit of herbicides in water is 70 μg L^−1^. In terms of herbicide capture, MOFs show superior performance when compared with their contemporary materials, mostly because of their high structural tuneability, porosity and chemical resistance in various conditions. For these reasons, some well-known MOFs like MIL-53(Cr) showed a high adsorption capacity of 556 mg g^–1^ for 2,4-dichlorophenoxyacetic acid, a well-known herbicide [95]. The surface charge on MOFs plays a crucial role in such capture processes due to the electrostatic attraction to some of the charged herbicide moieties. Methylchlorophenoxypropionic acid (MCPP), another well-known herbicide, was captured by UiO-66 up to 370 mg g^−1^, which was far superior to activated carbon. Electrostatic interaction and π – π interactions were mostly attributed to capture performance, which was governed by the pseudo-second-order nonlinear kinetic model [96]. Cationic herbicides like methyl viologen (MV) or paraquat (PQ) and diquat (DQ) have been captured by the effects of molecular sieving and fluorescence quenching, which are prevalent in the MOF domain. The Ni-PTC MOF, developed by Hill and co-workers, showed the molecular sieving effect in the capture of methyl viologen [97].

### Photocatalytic degradation of toxic organic pollutants

As a result of rapid urbanization and industrialization, several hazardous organic pollutants such as dyes, PPCPs, inflammatory drugs, and phenolic- and furan-based pollutants constantly contaminate the ground water. Photocatalytic degradation of such pollutants using MOFs has evolved as one of the most efficient methods of decontaminating waste-water sources. Semiconducting MOFs, which have large surface area and dense active sites, serve as the ideal photocatalytic materials for degradation of such organic pollutants. Several dyes, like rhodamine B (RhB), methylene blue (MB) and methyl orange (MO), have been the target analytes and have been degraded by MOFs over the past several years. The mechanism of such photocatalytic degradation of dye molecules is based on the absorption of visible light by MOFs, resulting in the formation of radicals (OH and O_2_) that degrade the dyes into cheap fuels like CO_2_ and H_2_O. PCN-224, composed of porphyrin-based ligands, shows the highest capacity of absorption of MO. The interaction between the incoming dye molecules, the Zr_6_ cluster and the porphyrin linker in this MOF, via π−π interactions and hydrogen bonding, results in the facile capture of dye molecules and subsequent degradation in visible light [98]. The introduction of Ti metal cations into the UiO-66 MOF has been used to create photocatalytic activity. The oxo-bridged hetero-Zr-Ti clusters result in 87% removal and degradation of MB under visible light.

Phenolic pollutants, viz. phenol, naphthol, chlorophenol and nitrophenol, have long been major sources of contamination for soil, groundwater and various other anthropogenic sources. Naphthol, which has a toxic effect on human blood circulation, was degraded up to 98.9% by ZIF-67@ZIF-8@MIL-125-NH_2_ (Fig. 7b and c) [99]. The degradation rate was ∼40%–50% higher than ZIF-67@MIL-125-NH_2_ and ZIF-8@MIL-125-NH_2_. In another work, Pt@UiO-66-NH_2_ was prepared by dispersing Pt onto UiO-66-NH_2_. A photocatalytic reactor was further designed based on this composite material using Al_2_O_3_ support, and 70% of phenol was degraded within 300 min of simulated sunlight. A dichlorophenol and trichlorophenol core-shell heterojunction in a MOF composite based on NH_2_-MIL-125 and Bi_2_MoO_6_ improved the charge separation, resulting in 93.28% and 92.19% degradation of dichlorophenol and trichlorophenol respectively (Fig. 7d) [100].

Some other organic pollutants, such as anti-inflammatory drugs like ibuprofen, ketoprofen, ofloxacin and tetracycline, have also been degraded photocatalytically by MOF-based catalysts. Several Ti-, Zr- and ZIF-based MOFs and MOF composites have shown attractive performances with regard to the effective degradation of such non-steroidal anti-inflammatory drug molecules at low irradiation time, with excellent kinetics. The presence of active sites in addition to several molecular interactions results in these degradation processes. Table 3 summarizes the performance of MOFs with regard to the photocatalytic degradation of inflammatory drugs.

**Table 3.** Performance of MOFs for inflammatory drug degradation by photocatalysis.

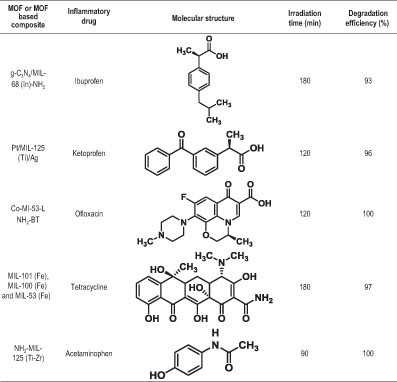

## CONCLUSION AND FUTURE OUTLOOK

In this review article we discuss the prospects of MOFs as efficient sensors and scavengers for environmental pollutants (neutral, cationic and anionic species). MOFs, due to their unique structure and composition tunability, pore architectures, and physiochemical stability, have been a forerunner in the material science field for the remediation of various environmental pollutants. Most of the toxic/hazardous pollutants have a direct adverse effect on human health, so it is crucial to monitor, detect and sequester them from various anthropogenic sources. Over the years, the WHO, the Environmental Protection Agency and various other international bodies have stressed the fact that production of these toxic pollutants must be controlled for a sustainable future and a clean, green environment. These pollutants are mostly found in aquatic bodies, such as industrial waste water, sewage and storm water, and affect humans and other animal species. Countries where rapid urbanization, technological development and globalization have taken place are most affected by environmental pollution. It is up to the scientists and/or engineers to come up with groundbreaking solutions to address this global problem. MOFs can indeed play an important role in this endeavour, as their porous architectures coupled with tunable functional units make them ideal candidates to trap and detoxify perilous entities from air and water. The remarkable structural diversity, high porosity and easy functionalizability, as well as excellent physiochemical stability, of MOFs set them apart from other contemporary materials, including zeolites, organic polymers and carbon nanotubes (CNTs). The principles of sensing, capture, degradation and detoxification of toxic entities mostly rely on the creation of highly selective functional/recognition sites via various modifications of MOF sensors. Once the MOF materials are optimized at the laboratory level for the detection and sequestration of toxic species, the next step would be to scale up for industrial and real-time application. Several engineering issues must be taken care of, including, but not limited to, material processability, efficiency and material costs, which could ultimately make MOFs the perfect candidates for environmental pollution remediation—a global challenge of high importance.
